# Usefulness of metabolic syndrome score in the prediction of angiographic coronary artery disease severity according to the presence of diabetes mellitus: relation with inflammatory markers and adipokines

**DOI:** 10.1186/1475-2840-12-140

**Published:** 2013-10-02

**Authors:** Jong-Youn Kim, Eui-Young Choi, Hee-Sun Mun, Pil-Ki Min, Young-Won Yoon, Byoung Kwon Lee, Bum-Kee Hong, Se-Joong Rim, Hyuck Moon Kwon

**Affiliations:** 1 Cardiology Division, Department of Internal Medicine, Gangnam Severance Hospital, Yonsei University College of Medicine, 211 Eonju-ro, Gangnam-gu, Seoul 135-720, Korea; 2 Severance Institute for Vascular and Metabolic Research, Yonsei University College of Medicine, Seoul, Korea

**Keywords:** Metabolic syndrome, Adipokines, Coronary artery disease, Diabetes mellitus

## Abstract

**Background:**

It is a matter of debate whether metabolic syndrome (MS) improves cardiovascular risk prediction beyond the risk associated with its individual components. The present study examined the association of MS score with high sensitivity C-reactive protein (hs-CRP), interleukin-6 (IL-6), resistin, adiponectin, and angiographic coronary artery disease (CAD) severity according to the presence of DM. In addition, the predictive value of various clinical and biochemical parameters were analyzed, including the MS score for angiographic CAD.

**Methods:**

The study enrolled 363 consecutive patients (196 men, 62 ± 11 years of age) who underwent coronary angiography for evaluation of chest pain. Blood samples were taken prior to elective coronary angiography. MS was defined by the National Cholesterol Education Program criteria, with MS score defined as the numbers of MS components. CAD was defined as > 50% luminal diameter stenosis of at least one major epicardial coronary artery. CAD severity was assessed using the Gensini score.

**Results:**

Of the 363 patients studied, 174 (48%) had CAD and 178 (49%) were diagnosed with MS. When the patients were divided into 4 subgroups according to MS score (0–1, 2, 3, 4–5), IL-6 levels and the CAD severity as assessed by the Gensini score increased as MS scores increased. In contrast, adiponectin levels decreased significantly as MS scores increased. When subjects were divided into two groups according to the presence of DM, the relationships between MS score and IL-6, adiponectin, and Gensini score were maintained only in patients without DM. Age, smoking, DM, MS score, and adiponectin independently predicted angiographic CAD in the whole population. However, age is the only predictor for angiographic CAD in patients with DM.

**Conclusions:**

In the presence of DM, neither adipokines nor MS score predicted angiographic CAD. However, in non-diabetic patients, IL-6 and adiponectin showed progressive changes according to MS score, and MS score was an independent predictor of CAD in patients without DM.

## Background

Metabolic syndrome (MS) is considered a clinical predictor of cardiovascular disease [1-3], and patients with MS have a higher incidence of coronary artery disease (CAD) than individuals without MS [4,5]. Visceral adipose tissue plays a crucial role in the pathogenesis of MS [6]. Adipokines, which are bioactive derivatives produced by the adipose tissue, may contribute to the pathogenesis of cardiovascular disease in patients with MS. [7,8].

However, it is still debatable whether the CAD risk associated with MS is above and beyond the risk associated with its individual components [9,10]. Previous studies have reported that the number of markers of MS, or the MS score, is more useful than a binary definition of MS to predict severity of CAD [11,12]. Moreover, the predictability of MS for CAD arises primarily from high fasting blood glucose [12] and the relationship between the MS score and CAD severity is unclear in the presence of diabetes mellitus (DM) [13].

To the best of the author’s knowledge, the effect of DM on the relationship between MS score and levels of inflammatory markers or adipokines has not been investigated. Therefore, the present study examined the association of MS score with high sensitivity C-reactive protein (hs-CRP), interleukin-6 (IL-6), resistin, adiponectin, and angiographic CAD severity according to the presence of DM. In addition, this study analyzed the predictive value of various clinical and biochemical parameters, including MS score, for angiographic CAD.

## Methods

### Subjects

The study prospectively enrolled 363 consecutive patients who underwent their first elective coronary angiography for evaluation of chest pain from October 2007 to June 2008 at the Gangnam Severance Hospital, Yonsei University College of Medicine, Seoul, Korea. Patients who had a history of previous percutaneous coronary intervention or coronary artery bypass graft were excluded. Other exclusion criteria were acute myocardial infarction, apparent infectious disease, chronic kidney disease, chronic inflammatory disorders, and malignancy.

The study protocol was approved by the Institutional Review Board of the Gangnam Severance Hospital, Yonsei University College of Medicine, and a written informed consent was obtained from each patient.

### Definition of metabolic syndrome

The presence of MS was determined using the updated 2005 Third Adult Treatment Panel of the National Cholesterol Education Program criteria [14]. In the present analysis, central obesity was considered to be present if waist circumference was ≥ 90 cm in men and ≥ 80 cm in women, using thresholds for the Asian population. Those who had any three or more of the five components were classified as having MS. The MS score was defined as the number of constituents of MS.

### Biochemical assessment

Blood samples were taken in the early morning after overnight fasting prior to elective coronary angiography. Serum lipid profiles, fasting blood glucose, serum insulin, and high sensitivity hs-CRP were measured in the hospital laboratory. Insulin resistance was evaluated in non-diabetic patients by the homeostatic model assessment as previously described [15].

Blood samples for analysis of IL-6, resistin, and adiponectin were collected through the vascular sheath prior to angiography. Serum was separated and stored frozen at −80°C for subsequent assay. Serum resistin and IL-6 were measured by enzyme-linked immunosorbent assay using commercial kits (R&D Systems, Minneapolis, MN, USA). Serum adiponectin levels were measured using a commercially available radioimmunoassay (Linco Research, St. Charles, MO, USA).

### Angiographic assessment

Coronary angiography was performed by conventional methods via the femoral artery. The angiographic characteristics of all coronary lesions in the index coronary angiogram were obtained by carefully reviewing the angiogram. CAD was defined as > 50% luminal diameter stenosis of at least one major epicardial coronary artery. CAD severity was assessed using the Gensini scoring system as previously described [16]. Grades of luminal stenosis were determined by consensus opinion of two experienced interventional cardiologists.

### Statistical analyses

Continuous data are expressed as the mean ± S.D., and categorical data are presented as numbers and percentages. Differences in categorical variables were analyzed using the chi-square test, and continuous variables were analyzed using the Student’s t-test. Comparisons among groups according to MS score were calculated with an analysis of variance for continuous variables. Multivariate logistic regression analysis was performed to determine predictors of angiographic CAD. The base-2 logarithms (log_2_) of the levels of hs-CRP, IL-6, resistin, and adiponectin were used in logistic regression analysis to account for skewed distribution [17,18]. Thus, odds ratios for these parameters reflect the change in odds for an increase of 1 log_2_ (the equivalent of a doubling of the value) in the measure. A 2-tailed *p* value < 0.05 was considered statistically significant. All statistical analyses were performed with PASW statistics version 18.0 (SPSS, Inc., Chicago, IL, USA).

## Results

A total of 363 patients (196 men, 62 ± 11 years of age) were enrolled in this study. Of these 363 patients, 174 (48%) had CAD and 178 (49%) were diagnosed with MS. The distribution of patients with an MS score of 0 to 5 is presented in Table 1. In most groups, high blood pressure was the most frequent abnormality, followed by increased waist circumference, low high-density lipoprotein cholesterol, high fasting blood glucose, and high triglycerides.

**Table 1:** Relative frequency of components of metabolic syndrome and clinical characteristics according to metabolic syndrome score

**Parameters**	**MS scores**				**Total(n = 363)**
	**0,1 (n = 95)**	**2 (n = 90)**	**3 (n = 104)**	**4,5(n = 74)**	
High BP^*^	17 (18%)	57 (63%)	87 (84%)	68 (92%)	229 (63%)
High FBG^†^	4 (4%)	19 (21%)	44 (42%)	50 (67%)	117 (32%)
Low HDL-C^‡^	17 (18%)	38 (42%)	70 (67%)	68 (92%)	193 (53%)
High TG^§^	1 (1%)	13 (14%)	43 (41%)	60 (81%)	117 (32%)
Abdominal obesity^║^	23 (24%)	53 (59%)	68 (65%)	68 (92%)	212 (58%)
Age (years)	61 ± 11	63 ± 11	63 ± 11	61 ± 10	62 ± 11
Male	53 (56%)	47 (52%)	57 (55%)	39 (53%)	196 (54%)
Smoking	14 (15%)	14 (16%)	14 (14%)	8 (11%)	50 (14%)

MS = metabolic syndrome; BP = blood pressure; FBG = fasting blood glucose; HDL-C = high-density lipoprotein cholesterol; TG = triglyceride.

^*^> 130/85 mmHg.

^†^ ≥ 110 mg/dL.

^‡^For men < 40 mg/dL; for women < 50 mg/dL.

^§^≥ 150 mg/dL.

^║^For men, waist circumference > 90 cm; for women, waist circumference > 80 cm.

Demographic and biochemical characteristics of patients with and without MS are presented in Table 2. Compared to patients without MS, patients with MS had higher serum resistin levels (7.1 ± 7.0 vs. 5.8 ± 3.8 ng/mL, *p* = 0.036) and lower serum adiponectin levels (5.8 ± 4.7 vs. 7.5 ± 5.7 μg/mL, *p* = 0.002). The levels of hs-CRP and IL-6 were not significantly different between the groups with and without MS. The homeostasis model assessment of insulin resistance calculated for non-diabetic patients (n = 276) was higher in patients with MS (2.5 ± 3.5 vs. 1.4 ± 1.7, *p* < 0.001).

**Table 2:** Clinical and biochemical characteristics according to the presence of metabolic syndrome

**Parameters**	**Without MS (N = 185)**	**With MS (N = 178)**	***p*value**
Age (years)	61.8 ± 11.5	62.2 ± 10.6	0.728
Male	100 (54%)	96 (54%)	0.981
BMI (kg/m^2^)	23.7 ± 2.8	26.0 ± 2.8	<0.001
Waist circumference (cm)	85.9 ± 8.6	92.9 ± 9.2	<0.001
Hip circumference (cm)	91.2 ± 8.2	95.2 ± 7.0	<0.001
Waist-hip ratio	0.94 ± 0.07	0.97 ± 0.11	0.064
Systolic BP (mmHg)	122.9 ± 17.4	127.2 ± 20.2	0.028
Diastolic BP (mmHg)	74.2 ± 10.4	75.7 ± 12.0	0.189
Smoking	28 (15%)	22 (12%)	0.443
Hypertension	74 (40%)	155 (87%)	<0.001
Diabetes	17 (9%)	70 (39%)	<0.001
Total cholesterol (mg/dL)	161.3 ± 35.2	153.3 ± 41.8	0.050
TG (mg/dL)	97.8 ± 37.3	158.5 ± 78.2	<0.001
HDL-C (mg/dL)	48.4 ± 11.9	39.6 ± 8.5	<0.001
LDL-C (mg/dL)	101.7 ± 34.4	97.6 ± 30.9	0.245
Lipoprotein(a) (mg/dL)	22.4 ± 28.5	18.9 ± 21.5	0.196
FBG (mg/dL)	96.1 ± 18.0	106.6 ± 25.6	<0.001
Serum insulin (μIU/mL)	5.5 ± 4.9	9.4 ± 13.3	<0.001
HOMA-IR^*^	1.4 ± 1.7	2.5 ± 3.5	<0.001
hs-CRP (mg/L)	5.4 ± 13.7	6.1 ± 17.3	0.716
IL-6 (pg/mL)	2.3 ± 3.7	3.0 ± 5.0	0.103
Resistin (ng/mL)	5.8 ± 3.8	7.1 ± 7.0	0.036
Adiponectin (μg/mL)	7.5 ± 5.7	5.8 ± 4.7	0.002

MS = metabolic syndrome; BMI = body mass index; BP = blood pressure; TG = triglyceride; HDL-C = high-density lipoprotein cholesterol; LDL-C = low-density lipoprotein cholesterol; FBG = fasting blood glucose; HOMA-IR = the homeostatic model assessment of insulin resistance; hs-CRP = high sensitivity C-reactive protein; IL-6 = interleukin-6.

^*^HOMA-IR was calculated only for non-diabetic patients (n = 276).

The prevalence of angiographic CAD and multivessel disease is higher in patients with MS (Table 3). Moreover, patients with MS had more severe CAD as assessed by the Gensini score (20.7 ± 27.7 vs. 13.3 ± 20.4, *p* = 0.004). However, clinical presentation of CAD was not significantly different between the two groups.

**Table 3:** Clinical presentation, angiographic diagnosis, and severity of coronary artery disease according to presence of metabolic syndrome

**Parameters**	**Without MS (N = 185)**	**With MS (N = 178)**	***p*value**
UA	35 (19%)	46 (26%)	0.072
Angiographic CAD	76 (41%)	98 (55%)	0.008
MVD	36 (20%)	56 (32%)	0.009
Gensini score	13.3 ± 20.4	20.7 ± 27.7	0.004

MS = metabolic syndrome; UA = unstable angina; CAD = coronary artery disease; MVD = multi-vessel disease.

When patients were divided into four subgroups according to MS score, IL-6 levels and CAD severity assessed by Gensini score tended to increase as MS score increased (Table 4). Serum resistin tended to increase with MS score, but this trend was not significant. In contrast, adiponectin levels significantly decreased as MS score increased. However, hs-CRP was not significantly different among the four subgroups.

**Table 4:** Inflammatory markers, adipokines, and severity of angiographic coronary artery disease according to metabolic syndrome score

**Parameters**	**MS scores**				***p*value**
	**0,1(n = 95)**	**2(n = 90)**	**3(n = 104)**	**4,5(n = 74)**	
hs-CRP (mg/L)	5.8 ± 14.3	5.0 ± 13.1	5.2 ± 15.4	7.3 ± 19.8	0.827
IL-6 (pg/mL)	2.0 ± 2.8	2.6 ± 4.4	2.4 ± 3.5	4.0 ± 6.5	0.024
Resistin (ng/mL)	5.7 ± 4.1	6.0 ± 3.4	6.6 ± 5.0	7.8 ± 9.1	0.077
Adiponectin (μg/mL)	8.4 ± 6.7	6.6 ± 4.2	6.2 ± 5.1	5.3 ± 4.2	0.001
Gensini score	9.4 ± 16.8	17.4 ± 22.9	18.9 ± 24.8	23.1 ± 31.3	0.002

MS = metabolic syndrome; hs-CRP = high sensitivity C-reactive protein; IL-6 = interleukin-6.

**Figure 1:**
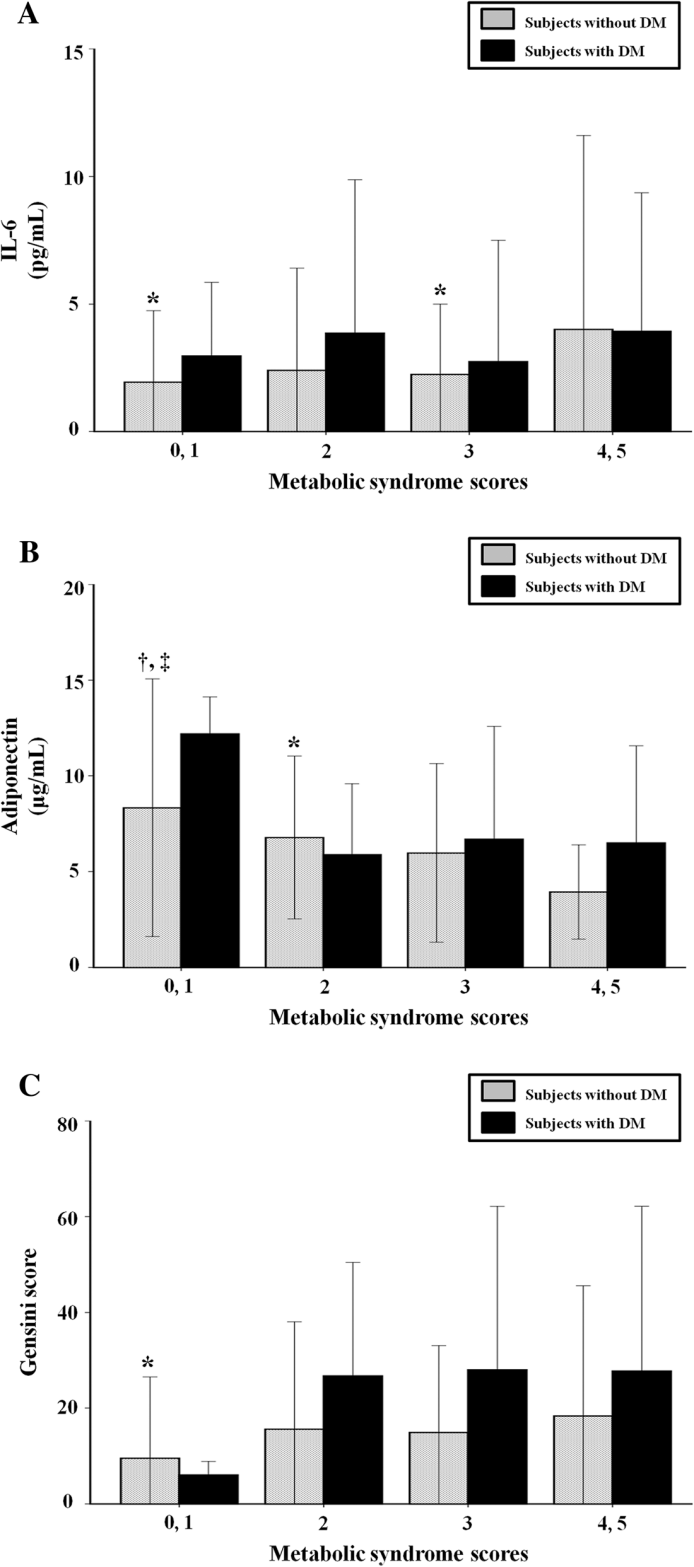
**Relation of metabolic syndrome score with interleukin-6 (A), adiponectin (B), and Gensini score (C) according to the presence of diabetes.** Data are expressed as mean ± standard deviation. DM, diabetes mellitus; IL-6, interleukin-6. ^*^p < 0.05 vs. metabolic syndrome (MS) scores 4, 5 group; †*p* < 0.05 vs. MS score 3 group; ‡*p* < 0.001 vs. MS score 4, 5 group.

When the subjects were divided into two groups according to the presence of DM, the relationships of IL-6, adiponectin, and Gensini score with MS score were maintained in patients without DM. In diabetic patients, however, there was no significant relationship between these parameters and MS score (Figure 1).

Various clinical and biochemical parameters including traditional CAD risk factors, inflammatory markers, adipokines, and MS score were analyzed for prediction of angiographic CAD. When a multivariate logistic regression analysis was performed, age, smoking, DM, MS score, and adiponectin independently predicted angiographic CAD in the entire population (Table 5). These analyses were then performed in subgroups according to presence of DM. Age, smoking, and MS score were still independent predictors for angiographic CAD in patients without DM. However, age is the only predictor for angiographic CAD in patients with DM (Table 6).

**Table 5:** Multiple clinical and biochemical parameters as determinants of angiographic coronary artery disease in multivariate logistic regression analysis

**Parameters**	**Odds ratio (95% CI)**	***p*value**
Age (per yr)	1.046 (1.018 – 1.074)	0.001
Smoking (yes)	4.155 (1.763 – 9.791)	0.001
Hypertension (yes)	0.661 (0.338 – 1.292)	0.661
Diabetes mellitus (yes)	2.290 (1.166 – 4.499)	0.016
Dyslipidemia (yes)	1.507 (0.746 – 3.045)	0.253
MS score (per 1 point)	1.452 (1.109 – 1.901)	0.007
hs-CRP (per doubling)	1.052 (0.862 – 1.284)	0.619
IL-6 (per doubling)	1.064 (0.781– 1.450)	0.695
Resistin (per doubling)	1.023 (0.669 – 1.565)	0.916
Adiponectin (per doubling)	0.683 (0.469 – 0.995)	0.047

MS = metabolic syndrome; hs-CRP = high sensitivity C-reactive protein; IL-6 = interleukin-6; CI = confidence interval.

**Table 6:** Multivariate regression analysis for the prediction of angiographic coronary artery disease in subgroups according to the presence of diabetes

	**Subjects without DM (n = 276)**		**Subjects with DM (n = 87)**	
**Parameters**	**Odds ratio (95% CI)**	***p*value**	**Odds ratio (95% CI)**	***p*value**
Age (per yr)	1.043 (1.012 – 1.075)	0.006	1.079 (1.003 – 1.161)	0.041
Smoking (yes)	3.843 (1.514 – 9.753)	0.005	9.428 (0.803 – 110.639)	0.074
Hypertension (yes)	0.659 (0.311 – 1.397)	0.277	0.442 (0.079 – 2.464)	0.352
Dyslipidemia (yes)	1.210 (0.547 – 2.680)	0.638	5.860 (0.625 – 54.969)	0.122
MS score (per 1 point)	1.461 (1.078 – 1.981)	0.015	1.598 (0.788 – 3.238)	0.193
hs-CRP (per doubling)	1.066 (0.852 – 1.333)	0.577	0.929 (0.578 – 1.492)	0.759
IL-6 (per doubling)	1.087 (0.760 – 1.555)	0.648	1.063 (0.557 – 2.030)	0.853
Resistin (per doubling)	0.990 (0.610 – 1.609)	0.969	1.195 (0.469 – 3.043)	0.709
Adiponectin (per doubling)	0.646 (0.417 – 1.001)	0.051	0.985 (0.424 – 2.290)	0.972

MS = metabolic syndrome; hs-CRP = high sensitivity C-reactive protein; IL-6 = interleukin-6; DM = diabetes mellitus; CI = confidence interval.

## Discussion

MS is defined as the presence of any three or more of five quantitatively identified markers. Although the individual components of MS are related to one another, patients with MS could be comprised of heterogeneous subgroups [12]. Therefore, it has been a matter of debate whether MS improves cardiovascular risk prediction beyond the risk associated with its individual components [9,10]. Some studies have reported that MS score is more useful than the presence or absence of MS in predicting the severity of CAD [11,12]. Moreover, it has been suggested that MS predicts CAD based primarily on high fasting blood glucose [12], and the relationship between the MS score and CAD severity is unclear in the presence of DM [13].

The present study demonstrated that incremental changes in angiographic CAD severity assessed by the Gensini score were observed in accordance with MS score, and MS score was an independent predictor for angiographic CAD. However, in a diabetic subgroup, this association was not observed, and MS score could not predict CAD. These results are consistent with previous findings [13,19]. This study expands upon existing reports by demonstrating that MS scores are significantly related to IL-6 and adiponectin levels in the non-diabetic subgroup, but not in the diabetic subgroup. Regarding the prediction of angiographic CAD, adiponectin showed marginal significance only in the non-diabetic subgroup [20].

Although the pathophysiological mechanism by which MS increases cardiovascular risk is still unclear [21], insulin resistance and central obesity seem to be essential components of MS [22,23]. Obesity as a predictor of cardiovascular events is related to many cardiovascular risk factors which are also components of MS [21,24]. It has been shown that visceral adipose tissue is an active endocrine organ which produces several bioactive derivatives including proinflammatory and prothrombotic adipokines, and protective adiponectin [25]. Visceral fat accumulation followed by increased production of proinflammatory adipokines and decreased production of adiponectin is associated with individual components of MS such as insulin resistance, hypertension, and dyslipidemia [6]. Eventually, these abnormalities could lead to atherosclerosis and cardiovascular events [26,27].

Some experts have suggested that increased cardiovascular risk associated with MS primarily arises from the presence of DM, and DM should be excluded from the definition of MS [28]. The results of the present study also suggest that neither adipokines nor MS score have an incremental value for the prediction of CAD in the presence of DM. As DM is a very strong predictor of cardiovascular disease, the five components of MS and other non-traditional markers of cardiovascular disease did not improve cardiovascular disease prediction beyond the contribution of DM [29].

The definition of MS excludes other factors related to insulin resistance such as proinflammatory adipokines or adiponectin. It is unclear whether inclusion of these components would predict cardiovascular disease better than the current components [28,30,31]. However, in the non-diabetic subgroup, IL-6 and adiponectin changed gradually according to MS score, and MS score predicted angiographic CAD. Therefore, MS score could be used as a predictor of CAD in subjects without DM. The gradual change of inflammatory markers or adipokines according to MS score might be at least partly related with the usefulness of MS score in the prediction of CAD in non-diabetic patients.

The present study has several limitations. Firstly, the subjects in this study were patients who underwent coronary angiography for clinically suspected CAD. Therefore, the prevalence of DM (24%) or CAD (48%) was quite high, and selection bias may affect the results. Secondly, the severity of CAD was assessed by the Gensini scoring system based on the degree of angiographic luminal stenosis. Thus, lesion complexity or plaque vulnerability could not be analyzed in the present study. However, angiographic assessment of coronary stenosis is a widely accepted and clinically useful method for risk stratification in CAD patients. Lastly, this was a cross-sectional study and therefore it was not possible to determine causative relationships. In addition, the effects of medications that could influence the levels of inflammatory cytokines and adipokines, including anti-diabetic drugs, could not be fully analyzed in this study. Therefore, we cannot rule out completely the possibility that these medications might influence the results in some cases.

## Conclusions

In the presence of DM, neither adipokines nor MS score had incremental value for predicting angiographic CAD. However, in non-diabetic patients, IL-6 and adiponectin gradually changed according to the MS score, and MS score predicted angiographic CAD. Therefore, MS score could be a useful predictor of CAD in patients without DM.

## Abbreviations

MS: Metabolic syndrome; CAD: Coronary artery disease; DM: Diabetes mellitus; hs-CRP: High sensitivity C-reactive protein; IL-6: Interleukin-6; Log2: Base-2 logarithms.

## Competing interests

The authors declare that they have no competing interests.

## Authors’ contributions

KJY, MPK, RSJ, and KHM participated in the design, coordination, and interpretation of this study. CEY and MHS contributed to the collection of clinical and laboratory data. YYW, LBK, and HBK contributed to the collection and analysis of angiographic data. KJY and MPK wrote this manuscript. All authors read and approved the final manuscript.
